# ENERDGE: Distributed Energy-Aware Resource Allocation at the Edge

**DOI:** 10.3390/s22020660

**Published:** 2022-01-15

**Authors:** Marios Avgeris, Dimitrios Spatharakis, Dimitrios Dechouniotis, Aris Leivadeas, Vasileios Karyotis, Symeon Papavassiliou

**Affiliations:** 1 Department of Electrical and Computer Engineering, National Technical University of Athens, 15780 Athens, Greece; dspatharakis@netmode.ntua.gr (D.S.); ddechou@netmode.ntua.gr (D.D.); papavass@mail.ntua.gr (S.P.); 2 École de Technologie Supérieure, Montreal, QC H3C 1K3, Canada; aris.leivadeas@etsmtl.ca; 3 Department of Informatics, Ionian University, 49100 Corfu, Greece; karyotis@ionio.gr

**Keywords:** task offloading, edge computing, energy optimization, resource allocation, Markov Random Fields

## Abstract

Mobile applications are progressively becoming more sophisticated and complex, increasing their computational requirements. Traditional offloading approaches that use exclusively the Cloud infrastructure are now deemed unsuitable due to the inherent associated delay. Edge Computing can address most of the Cloud limitations at the cost of limited available resources. This bottleneck necessitates an efficient allocation of offloaded tasks from the mobile devices to the Edge. In this paper, we consider a task offloading setting with applications of different characteristics and requirements, and propose an optimal resource allocation framework leveraging the amalgamation of the edge resources. To balance the trade-off between retaining low total energy consumption, respecting end-to-end delay requirements and load balancing at the Edge, we additionally introduce a Markov Random Field based mechanism for the distribution of the excess workload. The proposed approach investigates a realistic scenario, including different categories of mobile applications, edge devices with different computational capabilities, and dynamic wireless conditions modeled by the dynamic behavior and mobility of the users. The framework is complemented with a prediction mechanism that facilitates the orchestration of the physical resources. The efficiency of the proposed scheme is evaluated via modeling and simulation and is shown to outperform a well-known task offloading solution, as well as a more recent one.

## 1. Introduction

The proliferation of telecommunications in the last decade has offered a plethora of new applications and features to the end-users. End-devices with cameras, navigation systems, and embedded sensors support various augmented capabilities, while the introduction of new communication and network paradigms, such as the Internet of Things (IoT) and 5G networks, have resulted in an exponential increase of generated traffic volume and order of end-devices in wireless networks.

Although the evolution of wireless communications is accompanied with computationally powerful devices, applications still need to fully or partially offload the involved computational tasks. The reason is that mobile applications are becoming more complex and more demanding in terms of Quality of Service (QoS) and Quality of Experience (QoE) [1,2]. An efficient way to enable task-offloading and energy savings is to leverage the abundant resources available in the Cloud. This mobile-to-Cloud interconnection can facilitate the execution of computationally-intensive and data-driven processing tasks in a relatively low-cost and effective manner [3]. However, the use of Cloud Computing (CC) for task offloading of the end-devices can generate two major issues: high transmission latency and capacity-demand mismatch, i.e., resource overprovisioning, which leads to resource and energy waste [4]. To mitigate this, the Edge Computing (EC) approach, which pushes computing capabilities at the Edge of the network, is being rapidly adopted and seems promising in terms of achieving the ambitious millisecond-scale latency required in various 5G and IoT applications [5].

### 1.1. Motivation & Challenges

However, despite the numerous possibilities and advantages introduced by EC—in contrast with the Cloud where large-scale computational and communication infrastructures are the norm—the resources at the Edge are limited to micro data-centers, consisting of only few servers [6]. Thus, an efficient resource allocation technique is required for both users and infrastructure providers. On the user side, task offloading aims to respect the latency constraints and extend the battery lifetime. The success of task offloading depends mainly on the user’s mobility and the quality of wireless connection [1]. On the provider side, the primary goal is the minimization of the energy consumption of the data center, which is mainly affected by the number of active servers and the amount of their allocated resources [7,8]. Thus, task offloading and resource allocation are coupled and must be jointly addressed.

To this end, a synergistic and distributed approach between the end-devices and the edge infrastructure is necessary to accommodate the dynamic demand of the applications. The main challenge of such an approach is to estimate the amount of the offloaded tasks and make appropriate decisions on where the offloaded tasks should be executed. Taking into consideration the wireless channel conditions, the complexity of this resource allocation problem increases exponentially. Dynamic physical channel conditions and dynamic user density, due to users’ mobility in the infrastructure, require a proactive and dynamic resource allocation technique to select the necessary computational and networking resources at the Edge, in an adaptive manner. This creates the need to investigate appropriate resource allocation strategies enhanced with user density prediction techniques, to further ameliorate the delay and energy savings of both end-devices and edge infrastructure.

### 1.2. Contributions & Outline

In order to satisfy the aforementioned requirements, we propose a novel framework, referred to as ENERDGE, which jointly tackles task offloading and resource allocation of multiple edge data centers in a distributed and energy-efficient manner. The framework has a gradual operation, introducing the following key contributions:

- We propose a performance modeling approach based on Switching Systems Theory, to define virtual hardware profiles, i.e., flavors, for the edge infrastructure, providing application QoS guarantees under various operating conditions. The specific QoS metric investigated in the proposed approach is the application’s response time, but other relevant metrics could have been used as well. This modeling allows for dynamic selection and allocation of the appropriate amount of resources for each application (i.e., switching between the different hardware profiles), based on the anticipated workload demands. Leveraging the capabilities provided by this switching, we design a two-stage distributed, energy aware, proactive resource allocation mechanism.
- During the first stage, we extend current literature works that jointly address task offloading and resource allocation on a single edge site (i.e., [9]), to simultaneously minimize the total energy consumption of each edge site and provide guaranteed satisfaction of the QoS requirements of each deployed application. In order to accommodate the workload prediction demands at this stage, we utilise an existing user mobility prediction mechanism, based on the concept of the *n*-Mobility Markov Chain location prediction [10], to estimate the movement of the mobile devices between different sites within the edge infrastructure and subsequently the density of the users on each point of interest.
- During the second stage, we combine this approach with a novel Markov Random Field (MRF) mechanism that incorporates in its objective function all optimization criteria; this mechanism aims at redirecting tasks that cannot be executed locally under the given energy and QoS requirements of the first step, balancing resource utilization throughout the whole infrastructure. Thus, it achieves a better total energy management optimization through an efficient state space search in a distributed fashion, while taking into consideration any additional network delays incurred. This is the first approach of such a combination, and it could potentially pave the way for other similar MRF designs as optimizers in relevant problems. The integration of the above modeling and resource allocation approaches composes a task offloading and energy-aware resource allocation mechanism for accommodating dynamic spatiotemporal workload demands.
- Finally, we provide a detailed evaluation of our approach in terms of energy consumption minimization and QoS satisfaction for both stages of the mechanism. Then, we compare it with a well-established study [11] and a more recent one [12]. Based on a realistic application simulation, our solution outperforms both approaches in terms of adaptation efficiency. In other words, our approach yields less energy consumption for achieving the same QoS guarantees, or equivalently, it achieves higher QoS guarantees for the same energy consumption.

The remainder of the paper is organized as follows: Section 2 provides a brief overview of the related literature. Section 3 provides the system model along with a high-level description of the introduced collaborative framework. In Section 4, the problem formulation and proposed solution for the problem at hand are presented in detail. In Section 5, a thorough evaluation of the proposed framework through modeling and simulation is presented. Finally, Section 6 concludes the paper and describes potential future work.

## 2. Related Work

The problem of task offloading falls into the knapsack resource allocation category which is NP-hard in general [13]. Most of the proposed approaches follow a partial or full offloading technique, according to whether the tasks are separated or not, with the goal to minimize the overall latency and/or energy [14]. Furthermore, they propose static resource allocation schemes on the edge infrastructure. In this paper, we follow the design principles of [15] and propose the ENERDGE framework, a mobility-aware and full offloading approach in order to minimize the energy consumption of the edge infrastructure under specific QoS guarantees for the mobile applications hosted. In this context, there are three interesting and related directions in the literature: (i) mobility prediction for task offloading, (ii) single-site task offloading and resource allocation, and (iii) multi-site task offloading and resource allocation.

### 2.1. Mobility Prediction for Task Offloading

The success of offloading decisions depends heavily on the dynamic nature of task behavior and user mobility. In particular, the users may move and resource prices for offloaded task execution may vary over time. This led the authors in [16] to propose an online algorithm with a logarithmic objective to minimize the resource usage of the edge infrastructure, while taking into account the impact of mobility in the latency. They also formulate a VM migration cost for the tasks that need to follow the users’ movement. A migration policy, however, for containers, is also formulated in [17], where the authors introduce an architecture in which Fog Computing services constantly move in order to be always close enough to the served IoT mobile devices. Utilizing neural networks and Markov chains, Labriji et al. [18] presented a mobility prediction algorithm to proactively and online migrate computation services (VMs) for vehicular 5G networks.

Since the mobility of the users can significantly impact the latency and increase the migration cost, the authors in [19] introduced a prediction mechanism to ameliorate the offloading performance. A similar approach is followed in [20], where the most popular services are proactively installed in the Edge servers located in the positions that the users will most probably visit, thus reducing the network delay during task offloading. Another approach, denoted as MAGA and introduced in [21], is based on frequent moving patterns of the users and a genetic algorithm to partially offload tasks to edge servers. However, in the preceding works, the authors assume static resource allocation at the edge, in terms of amount of resources utilized.

### 2.2. Single-Site Offloading & Resource Allocation

In case of task offloading, a single edge site is usually available in close proximity to the users. The main focus in this type of resource allocation problem lies in the latency and energy minimization. For example, the authors in [22] investigate the task offloading of augmented reality applications emphasizing on the computation intensive tasks (i.e., object recognition and position tracking). A successive convex approximation approach is proposed to minimize energy consumption under latency constraints, while emphasizing on both the available computation and communication resources at the Edge. Another energy-efficient based approach is presented in [13], following a mixed discrete-continuous optimization approach along with a low-complexity heuristic based on Johnson’s algorithm. Elgendy et al. [23] try to minimize the total consumed energy by solving an optimization problem to compute near-optimal offloading decisions for each mobile IoT user, however, for a single edge server and without considering the mobility of the users.

Regarding latency, authors in [4] study the admission control and resource allocation problem of computationally intensive IoT applications at the Edge. A Lyapunov dynamic stochastic optimization approach is used with the goal to reduce the end-to-end delay, while improving the overall throughput. Similarly, Ren et al. [24] investigated the mobile-edge computing offloading problem with the goal to minimize the latency in a multi-user scenario with joint communication and computational resources. The solution is based on the Lagrange multiplier method. However, such centralized task offloading approaches usually fail to apply to realistic scenarios of larger edge infrastructures with multiple, geographically distributed sites.

### 2.3. Multi-Site Offloading & Resource Allocation

In case of multiple edge sites in close proximity to the devices, task offloading includes both the resource allocation of the tasks and the selection of the right administrative domain (i.e., edge infrastructure). In this context, an edge orchestrator can be used to assign the tasks to the appropriate domain, with the goal to maximize the number of successfully assigned task requests [25]. Sonmez et al. [26] proposed a fuzzy workload orchestrator for multiple Edge and Cloud infrastructures. For each offloaded request, a set of fuzzy rules determined the destination computational unit within a hierarchical multi-site architecture. However, the authors empirically defined the fuzzy rule sets, while assuming static resource provisioning on the edge servers, which might not be applicable to real conditions where services typically bear different workload characteristics.

Another goal can be the balancing of the load between edge servers, while minimizing the application response time. In [11], over-utilized edge servers redirect part of their incoming workflow to resource-rich or under-utilized servers, using a minimum cost max flow algorithm towards achieving total balance in terms of average application response time in the whole edge infrastructure. An extension to this work is presented in [27], where a genetic algorithm is exploited for a distributed load balancing of traffic, yielding a solution that converges to the minimization of maximum task response time through gene mutations. A slightly different approach is followed in [12], where the authors developed a load balancing technique for distributed edge servers, using a game theoretic approach, and proposed a state-based distributed learning algorithm to obtain the optimal action at each reachable state. The existence of recurrent state Nash equilibrium was proven by using the potential game theory.

The ENERDGE framework simultaneously addresses energy consumption minimization and distributed load balancing, while respecting the applications’ QoS requirements. Initially, we simulate a wireless protocol to extract the instantaneous throughput under dynamic wireless network conditions, and we predict the density of the users around a point of interest, with the use of an *n*-Mobility Markov Chain location prediction method. Based on this prediction, we leverage pre-computed profiles of virtual machines (VMs) to enable proactive and dynamic resource allocation at each edge site, ensuring the QoS constraints of any deployed application. Containers can also be considered as the virtualization units without any change in the modeling. Finally, we introduce a novel load balancing technique based on Markov Random Fields (MRF) and load redirection, to appropriately redistribute the excess workload among the available edge sites, towards the minimization of the total energy consumption. To the best of our knowledge, this is the first research effort that takes into consideration holistically these task offloading objectives in distributed EC infrastructures.

## 3. System Model

### 3.1. Edge Infrastructure & Applications

**Figure 1:**
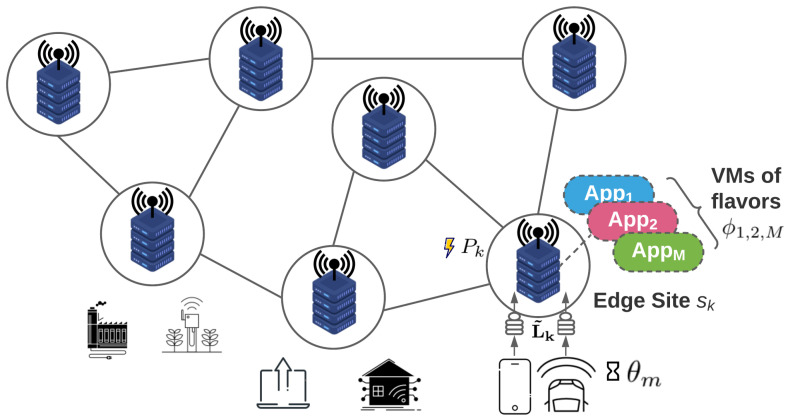
Example of Envisioned Edge Infrastructure.

To facilitate the extensive modeling employed in this work, Table 1 summarizes the key notation used throughout the article. We model our physical infrastructure as a group of wireless access points, each directly connected with a cluster of homogeneous servers, as illustrated in Figure 1. These physical resources altogether form an edge data center, which hereafter is referred to as site $s_{k}$, with $S={\left\{s_{k}\right\}}_{k=1}^{n}$ being the set of sites, for *n* sites in total. This set forms a graph, where each site corresponds to a node and the edges to the interconnections between them through routers, used only for forwarding purposes (i.e., backhaul network). Furthermore, we consider that the servers of the edge infrastructure located in different sites are heterogeneous. This implies differentiation on processing capabilities and service completion time among sites.

For the access layer, we assume the existence of various and heterogeneous end-devices (e.g., IoT, mobile devices) each associated with one of *M* specific mobile applications (i.e., augmented reality and wearables). Each application m∈{1,⋯,M} comes with specific requirements in terms of QoS (e.g., average response time) that will guide the allocation of the resources.

### 3.2. Task Offloading

As depicted in Figure 1, each end-device running an application *m* offloads its computational intensive processes to the Edge to reap the benefits of the more powerful computational resources. In this work, we assume an IEEE 802.11ac access network to offload the tasks from the devices. Following the work of [28], we model the access network using an indoor TGnAC Channel B, suitable for large open space and office environments [29]. Along the same lines, in order to capture the dynamic nature of the wireless channel, the transmission rate of the devices is adjusted according to an enhanced version of the Minstrel algorithm [30]. In this manner, the devices are able to change the modulation and coding scheme (MCS) used, and thus the transmission rate, conforming to the varying channel conditions and interference from nearby devices (Signal to Interference & Noise Ratio—SINR). This procedure allows us to create a realistic dataset containing tuples of *<number of users, offloading request rate of each user>*, which is publicly available (https://github.com/maravger/netmode-cloudsim/blob/master/task_offloading_ds_verbose.xlsx, accessed on 30 November 2021), and utilize it to translate the predicted number of users to the anticipated request rate, for a specific edge site. Specifically, we assume that each user constantly offloads at his/her maximum achievable data rate, and, considering a fixed offloaded task size, we are able to produce the anticipated workload volume for the estimated number of users.

We assume that each end-device needs to fully offload its requests on edge servers following a VM/container-based provisioning method. Depending on the user’s location, the offloaded tasks are initially assigned to the site where the wireless transmission occurs. Each VM/container of the site’s servers serves the offloaded requests of the application *m* that it was assigned to. We note here that, for the sake of simplicity, we focus on scenarios and settings where the user’s movement is typically limited close to the site of interest during the whole offloading procedure. Therefore, the offloading procedure for a single task is assumed to be completed within the same site that it was initiated in and, consequently, no handover processes and costs are considered. The most important QoS requirement of the offloaded tasks of an application *m* is the acceptable response time $\theta_{m}$ value, which is application-specific. Under this setting, the end-device accelerates the execution of computationally intensive tasks and extends its battery lifetime.

### 3.3. VM Flavor Design

On each edge site, it is essential to facilitate the proactive dynamic resource allocation due to the varying number of the offloading requests received. We denote the VM (or container) flavor for every deployed application, which describes the relation among the application’s response time, the allocated CPU cores, and the number of the offloaded requests. The computation of these VM flavors is based on switching systems from the System Theory. The advantage of the VM flavor design is two-fold; firstly, this modeling approach allows for accurately capturing the dynamic behavior of the application-specific VMs, under various operating conditions. Secondly, calculating a multitude of VM flavors allows us to quickly adjust the edge infrastructure to different pairs of workloads and applications, while providing a level of guarantee for the QoS specifications.

We define the VM (or container) flavor $\phi_{m}\in \Phi$ of an application *m* as a tuple that includes the QoS specifications of the hosted application, the requested resources for the VM that will provide the QoS guarantees and the maximum throughput of offloaded requests, for which the VM will be able to achieve these guarantees, $\phi_{m}:\;<\theta_{m},c_{m},\mu_{m}>$. Specifically, parameter $\theta_{m}$ denotes the average response time that the VM of flavor $\phi_{m}$ guarantees to achieve with $c_{m}$ CPU cores allocated to it and for a maximum throughput of $\mu_{m}$ offloaded requests per time unit. We assume that the response time consists of two terms: (a) transmission time and (b) service completion time. The transmission time includes the time to transmit/upload the application’s request through a wireless link. In particular, since we have modeled our wireless link through the IEEE 802.11ac protocol, we are able to calculate this delay by leveraging the information of throughput achieved and the application’s task size. Regarding, the time to download the response from the server, since the size of the output is generally much smaller than the input, this delay can be usually omitted [31]. Service completion time includes the VM/container startup time, as well as the queuing and processing time of the application tasks at the assigned servers. A flavor could also define the memory requested by the VM. However, it is omitted from the problem formulation due to the following reasons: Firstly, memory power consumption is negligible compared to CPU power consumption [32]. Secondly, following the paradigm set by well-known edge computing frameworks like MAUI [33] and ThinkAir [34], we concentrate on the offloading of CPU-intensive tasks.

In principle, the performance of an application hosted on a VM is nonlinear and cannot be described analytically. However, adopting linear modeling allows for an easier identification of the system, without significant loss of accuracy, and enables the implementation of various optimization and control methodologies. In order to extract the VM flavors for each application deployed on a site, we modify the modeling approach of [9]; for each application and for each flavor $\phi_{m}$ of this applications’ VMs, we identify a scalar, discrete Linear Time-Invariant (LTI) system. In particular, we mainly differentiate the VM flavors based on the number of CPU cores they require, which also constitutes the switching criterion of our mechanism. Thus, during this identification phase, for each application and for each different CPU core allocation, the operation of the corresponding VM is described by a discrete linear system of the following form:

(1) θ(τ+1)=aθ(τ)+bμ(τ),

where θ(τ) represents the average response time for the deployed application, within a time period τ and μ(τ) the number of offloaded requests within the specific time period. The coefficients a≥0 and b≥0 are known scalars which can be estimated by the Recursive Least Square algorithm [35].

Physically, a VM with $c_{m}$ allocated cores can only serve up to $\mu_{m}$ offloaded requests of the deployed application while guaranteeing an average response time of $\theta_{m}$ for the specific time period. This constitutes the physical interpretation of a flavor $\phi_{m}$ and generally, for each such switching system, a set of feasible VM flavors of this kind can be computed, according to certain performance criteria and input constraints. In our case, these feasible VM flavors are computed by solving the following linear programming problem with the goal to maximize the number of the offloaded requests:

(2a) $$\begin{array}{cc} \max_{\theta_{m},c_{m}}\;\; & \mu_{m} \end{array}$$

(2b) $$\begin{array}{cc} \mathrm{subject}\;\mathrm{to}\;\;\; & \theta_{m}=a\theta_{m}+b\mu_{m} \end{array}$$

(2c) $$\begin{array}{cc} \; & \theta_{\min}\leq \theta_{m}\leq \theta_{\max} \end{array}$$

(2d) $$\begin{array}{cc} \; & \mu_{\min}\leq \mu_{m}\leq \mu_{\max} \end{array}$$

The first constraint dictates that each flavor must also be an *equilibrium point of the discrete linear system*, which will guarantee its stability and confinement in a specific operating area around it. The second constraint implies that the average response time must lay between a minimum ($\theta_{\min}$) and a maximum value ($\theta_{\max}$), set by the application’s QoS requirements, while the last constraint refers to the offloaded requests varying within the applications anticipated throughput range. This problem is solved only once, in an offline manner, using the GLPK solver ([36]), thus its computational complexity is a fixed factor paid only once, at the very beginning of the operation of our framework. We do not consider it in the steady state of the framework’s operation, since it can be considered amortized in the long-run.

By having a set of VM flavors corresponding to different core allocations and maximum throughput, we provide a better level of accuracy than using a single LTI model for the whole operation. In such a way, the extracted VM flavors correspond to realistic operating conditions and constitute the fundamental elements for the ENERDGE resource allocation mechanism.

### 3.4. Power Modeling

When fully offloading tasks, the total computational and energy burden is shifted away from the devices. However, reviewing this shift from a complete network-wide view, one can easily understand that the problem is simply pushed at the Edge. Thus, in this work, we also consider the minimization of power consumption at the edge infrastructure. This includes switching physical devices on and off and optimizing the computational resource usage during the offloading.

Usually, for the server power dissipation, an almost linear relationship between the power consumption of a server and its CPU utilization exists. The following model can accurately predict the servers’ power consumption $P_{ser}$ with an error below 5% [32]:

(3) $$P_{ser}=\gamma \cdot P_{\max}+\left(1-\gamma \right)\cdot P_{\max}\cdot u,$$

where $P_{\max}$ is the maximum power consumed when the server is fully utilized, γ is the percentage of power consumed by an idle server (usually around 60% [37]), and *u* is the current CPU utilization.

In order to extract the power consumed by a VM of flavor $\phi_{m}$ (VM for application *m*) provisioned in a server, the above equation is transformed as follows:

(4) $$P\left(\phi_{m}\right)=\left\{\begin{array}{cc} \gamma \cdot P_{\max}+\left(1-\gamma \right)\cdot P_{\max}\cdot \frac{c_{m}}{Ser_{cpu}}, & \mathrm{if}\;u=0,\\ \left(1-\gamma \right)\cdot P_{\max}\cdot \frac{c_{m}}{Ser_{cpu}}, & \mathrm{otherwise}, \end{array}\right.$$

where $Ser_{cpu}$ is the total amount of the available computational resources in a server, i.e., CPU cores. Hence, for the first VM provisioned at a server, the power consumption will include activating the server and the power consumption added by the particular VM. For the rest of the VMs, only their power consumption is taken into consideration. It is worth mentioning that we assume an isolcpus technique [38], where we isolate and pin the requested CPU resources to the VM. This is a common technique for performance optimization when virtualizing x86 servers. Thus, each VM will have access only to its share of CPU resources consuming as well the corresponding power.

### 3.5. User Density and Workload Prediction

As discussed in the previous subsections, each site hosts a group of IoT/mobile applications and serves the offloaded requests that are generated by the devices within the range of its wireless access point. However, in both mobile and IoT applications, dynamic user density in the coverage area is a key feature and must be considered by the offloading decision and resource allocation mechanism, as it creates dynamic network conditions. Towards the optimal resource allocation policy, an accurate prediction of this is necessary.

In order to address this issue, we implement a variation of the *n*-Mobility Markov Chains (*n*-MMC) location prediction method described in [10]. In a nutshell, this method incorporates the two previous visited sites of a mobile device and a Mobility Markov Chain in order to probabilistically predict the device’s next location. As a prerequisite, this method requires a transition matrix available, containing all the feasible transitions of a device between the sites, associated with their probability of occurring.

In order to create this transition matrix, we used the Melbourne Museum dataset [39], which comprises 158 complete real visitor pathways, in the form of time-annotated sequences of visited exhibit sites. After processing the data, each path was assigned a probability based on its frequency of occurrence. This resulted in a transition matrix whose rows represent the three last visited sites and its columns represent the next site to be visited. In this way, predicting the next location of a visitor is simple. We trace their three most recently visited sites, search the row in the transition matrix that corresponds to this trace, and find the column with the maximum probability of transition for this row. The site of this column is the predicted next location. Finally, having available the collective statistics regarding the predicted locations of the users for the upcoming time period, we acquire the predicted offloaded workload, ${\tilde{L}}_{k}=\left[{\tilde{L}}_{k}^{m}\right]$, for the respective site $s_{k}$ and application *m*, as described in Section 3.2.

## 4. Resource Allocation & Workload Redistribution

Leveraging the Switching System modeling approach introduced in the previous section, in this section, we propose a 2-stage distributed, energy-aware, proactive resource allocation mechanism. In the first stage, an initial resource allocation optimization takes place locally at each site of the edge infrastructure, which balances between energy consumption minimization and QoS satisfaction. In the second stage, a novel distributed technique is applied to redirect the excess workload to under-utilized sites, thus balancing the resource utilization and achieving a better energy management.

### 4.1. Stage 1—Resource Allocation Optimization

In order to accommodate a proactive and dynamic resource allocation, we follow the work in [9] where time is considered slotted. In this stage, at the beginning of each system slot, a decision is made on the VM topology to be implemented on each site, which will enable it to handle the projected offloaded workload. This topology defines the number of edge servers to be activated in each site along with the VM formation to be placed in each edge server, i.e., the number and flavor of the VMs.

Feasible VM formations are the ones where the sum of the CPU cores requested from the co-hosted VMs’ flavors does not exceed a predefined threshold. For instance, assume two applications App1 and App2. A VM running App1 and instantiated in a flavor that requests two CPU cores, along with a VM running App2 and instantiated in a flavor that requests one allocated CPU core, is a feasible VM formation for a single edge server, as the cumulative number of allocated CPU cores does not exceed the threshold of three cores (75% of the server’s total available CPU capacity, $Ser_{cpu}=4$).

The set of all feasible VM formations for edge servers in site $s_{k}$ is defined as

(5) $${Ƶ}_{k}:=\left\{z_{i}=\left(\phi_{m}^{\left(j\right)},\ldots ,\phi_{m}^{\left(N\right)}\right),m\in \left[1,M\right],\;j\in \left[1,N\right]:\sum_{j=1}^{N}c_{m}^{\left(j\right)}\leq C_{k}^{ser}\right\},$$

where $i\in [1,|{Ƶ}_{k}|]$ is the index of the VM formation, $\phi_{m}^{\left(j\right)}$ is the VM flavor, $c_{m}^{\left(j\right)}$ the number of cores requested by the flavor of VM *j* of application *m*, *M* is the number of applications available at site $s_{k}$, *N* is the total number of VMs contained in formation $z_{i}$, and $C_{k}^{ser}$ is the CPU cores threshold set for each edge server of $s_{k}$. Due to the fact that the edge servers within a single site are considered homogeneous in terms of their resources, $C_{k}^{ser}$ has the same value for all of them that are tied to a site $s_{k}$.

We define the system cost as the power consumption of the edge infrastructure. Since in this stage of the resource allocation mechanism no exchange of workload takes place between the sites, minimizing locally the power consumption, $P_{k}$, of each individual site, $s_{k}$, results in minimizing the total power consumption, $P_{A}=\sum_{k=1}^{n}P_{k}$, where *n* stands for the total number of sites in the infrastructure. This can be achieved by optimizing the amount of edge resources that will be activated in each slot to serve the total predicted workload. Consequently, the corresponding optimization problem can be defined as:

(6a) $$\begin{array}{cc} \min_{f_{i},p_{i}}\;\; & \left\{P_{k}\right\} \end{array}$$

(6b) $$\begin{array}{cc} \mathrm{subject}\;\mathrm{to}\;\;\; & f_{i}\geq 0,\;i=1,\ldots ,\left|{Ƶ}_{k}\right| \end{array}$$

(6c) $$\begin{array}{cc} \; & \sum_{i=1}^{\left|{Ƶ}_{k}\right|}f_{i}\leq E_{k} \end{array}$$

(6d) $$\begin{array}{cc} \; & P_{k}=\sum_{i=1}^{\left|{Ƶ}_{k}\right|}f_{i}p_{i} \end{array}$$

(6e) $$\begin{array}{cc} \; & \sum_{i=1}^{\left|{Ƶ}_{k}\right|}f_{i}r_{i}^{m}\geq {\tilde{L}}_{k}^{m},\;\forall m\in \left\{1,\ldots ,M\right\}, \end{array}$$

where the positive integer variables $f_{i}$ denote how many servers need to be activated with the $z_{i}$ VM formation of set ${Ƶ}_{k}$, assuming the total number of formations of edge servers in site $s_{k}$ is $\left|{Ƶ}_{k}\right|$ and the total number of the available edge servers is $E_{k}$. Then, the sum of the $f_{i}$ variables cannot be greater than $E_{k}$ (constraint (6c)). Constraint (6d) requires that a site’s power consumption is equal to the sum of the power consumption of its activated edge servers.

As discussed in Section 3.4, the power consumption of each VM is proportional to its flavor size, i.e., the number of allocated CPU cores. As a result, power consumption $p_{i}$ of one edge server activated with the $z_{i}$ VM formation is calculated as follows:

(7) $$p_{i}:=p\left(z_{i}\right)=\sum_{j=1}^{N}P\left(\phi_{m}^{\left(j\right)}\right),\;m\in \left\{1,\ldots ,M\right\}.$$

Finally, the last *M* constraints of (6e) denote that the total predicted workload for each application at $s_{k}$, ${\tilde{L}}_{k}^{m}$, for the next system slot, is satisfied by the activated edge servers in each site. Again, as discussed in Section 3.3, the workload guaranteed to be served by one edge server with the $z_{i}$ VM formation is:

(8) $$r_{i}^{m}:=r^{m}\left(z_{i}\right)=\sum_{j=1}^{N}\mu_{m}^{\left(j\right)},\;m\in \left\{1,\ldots ,M\right\}.$$

**Figure 2:**
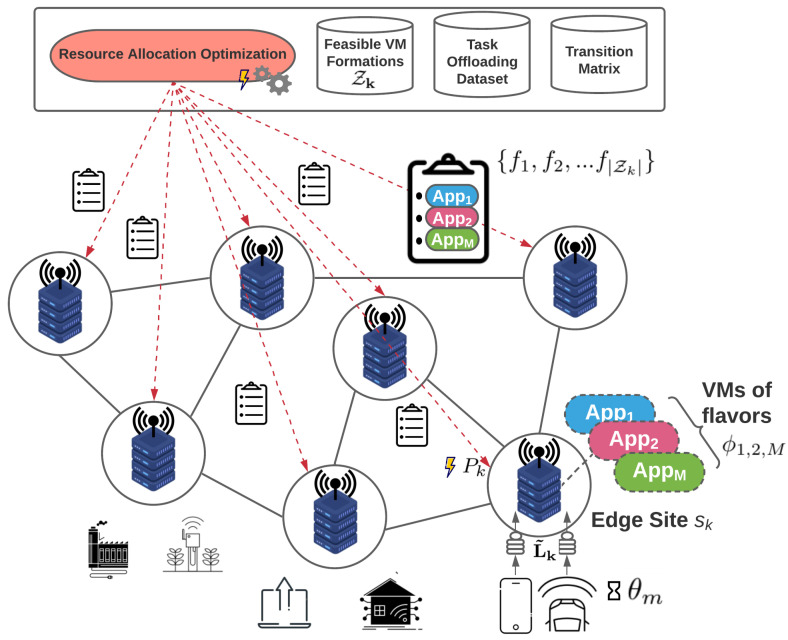
Resource allocation optimization overview (Stage 1).

Problem (6a) is solved in a distributed fashion, locally in each site and proactively at the beginning of each system slot, after collecting all the required information (i.e., available resources and predicted workload). An overview of this process is depicted in Figure 2. As evidenced by the above, the problem solved here is a combinatorial one, expressed as a mixed integer linear program (MILP). For treating this MILP, the GLPK solver is used once again. The problem under consideration is generally NP-hard, and the lower bound of the computational complexity of the branch-and-cut algorithm used to find a solution is exponential [40]. However, it should be noted that, following common considerations in the literature [9], we assume that the total number of available edge servers in a site is relatively small, thus the overall computation complexity of the optimization process is kept minimum, allowing the problem to be solved online.

### 4.2. Stage 2—Inter-Site Redistribution of Excess Workload

In edge infrastructures, the wireless network traffic, and therefore the offloading requests, exhibit considerable variation. On the one hand, there may be cases where the total predicted workload for a site exceeds its total available resources, in which case the problem in (6a) has no feasible solution. In this situation, all the site’s edge servers are activated with a fixed $z_{\max}$ formation, where $z_{\max}$ stands for the VM formation that accommodates the maximum possible number of offloaded requests for each application. Even so, a portion of the predicted workload will remain unserved (*overloaded site*). On the other hand, it is also common that the total predicted workload for a site is lower than the predefined threshold that dictates whether the energy cost of activating the site’s edge servers is worth serving it. Again, a portion of the predicted workload will remain unserved (*underloaded site*). We denote the aggregation of the remaining predicted workload of each of these sites as the *excess workload* $w_{k}$ of site $s_{k}$, and we handle this through the novel approach that follows.

In this second stage, we aim towards better balancing the previous resource management decisions, so that excess workload requests of a site are redistributed in neighboring (or even farther apart) sites. The excess workload is handled in such a way that it does not allow sites to become operational for a number of requests lower than a threshold of their total capacity, which will ensure eventually better energy efficiency, as explained in previous subsections. To achieve this, we employ the theory of Markov Random Fields (MRFs) [41], mainly due to their agile design and straightforward implementation, which allows simple distributed decision-making, while achieving results very close to the optimal ones (and frequently the optimal ones) with very low convergence times. The unfamiliar reader can refer to the Appendix A for a quick introduction to the MRF concept and basic notation.

In this work, we consider the sites $s_{k}\in S$. A neighborhood system $N={\left\{N_{s_{k}}\right\}}_{s_{k}\in S}$ is defined on *S*, while $N_{s_{k}}$ denotes the neighborhood of site $s_{k}$ and includes the nodes within single hop distance. Assume $w_{k}=\left[w_{m}^{\left(k\right)}\right]$ is the vector indicating the amount of excess workload for application *m* at each site $s_{k}$ and $b_{k}=\left[b_{i}^{\left(k\right)}\right]$ the vector indicating the number of selected servers of type *i*, to be additionally activated at site $s_{k}$. Considering $e_{k}$, the number of available servers per site $s_{k}$, which is obtained from the solution of the initial resource optimization problem (6a), $b_{k}$ is such that

(9) $$b_{k}=\left[b_{i}^{\left(k\right)},\ldots ,b_{\left|{Ƶ}_{k}\right|}^{\left(k\right)}\right],\;\sum_{i=1}^{\left|{Ƶ}_{k}\right|}b_{i}\leq e_{k}.$$

Vectors $w_{k},b_{k}$ are stochastic, since their values depend on the instantaneous system state and user activity. We define the collection of random variables $X_{k}={\left\{W_{k},B_{k}\right\}}_{k=1}^{n}$, as a collection of random vectors $W_{k}=w_{k},B_{k}=b_{k},\forall \;k\in \left[1,n\right]$, defining the state of each site and cumulatively the state of the system with respect to excess workload and available servers at each site $s_{k}$. The random field $X={\left\{X_{k}\right\}}_{k=1}^{n}$ takes values ${\left\{X_{k}=x_{k}\right\}}_{k=1}^{n}$ in Λ=W×B, which is the product space of phase spaces $w_{k}\in W,b_{k}\in B$, respectively. The configuration $\omega =\{x_{k}:x_{k}\in \Lambda ,\forall s_{k}\in S\}$ corresponds to one of all possible states of the system state and Λ denotes the configuration space.

Due to the distributed topology of the sites, the above random field X can be considered an MRF, and based on the Hammersley–Clifford theorem, we consider the potential function V(ω), which can be decomposed in clique potentials:

(10) $$V\left(\omega \right)=\sum_{C\in C}V_{C}\left(\omega \right)=\sum_{s_{k}\in S}V_{\left\{s_{k}\right\}}^{\left(1\right)}\left(\omega \right)+\sum_{s_{g}\in N_{s_{k}}}V_{\left\{s_{k},s_{g}\right\}}^{\left(2\right)}\left(\omega \right),$$

where C is the set of all cliques in the formed topology of sites (a clique denotes a subset of nodes, all of which are connected to each other). Depending on the characteristics of each topology, cliques of different sizes are formed and the potential function is computed over such cliques. The potential function is the objective function that we seek to minimize, and it will be used as a quantitative measure of the success of each system state to fulfil the optimization criteria, namely the reduction of the total power consumption of the Edge infrastructure. The lower the potential function, the more desired the corresponding system state will be. Due to the topology formed by the sites in this specific application (i.e., the access points), only one-clique (cliques consisting of one node— corresponding to the wireless access devices themselves) and two-cliques (cliques consisting of pairs only—pairs of wireless access devices) exist, so that the potential function is eventually decomposed in singleton $V_{\left\{s_{k}\right\}}^{\left(1\right)}\left(\omega \right)$ and doubleton (pairwise) $V_{\left\{s_{k},s_{g}\right\}}^{\left(2\right)}\left(\omega \right)$ terms, respectively. Each singleton term is defined as follows:

(11) $$V_{\left\{s_{k}\right\}}^{\left(1\right)}\left(x_{k}\right)=\left\{\begin{array}{cc} C_{1}\cdot P\left(b_{k}\right)\left[1+\sum_{m}\bar{\mathrm{sig}}\left(w_{m}^{\left(k\right)}\right)\right]+C_{2}\cdot d\cdot a_{k}, & \mathrm{if}\;\exists \;b_{k}\\ \; & \sum_{i=1}^{\left|{Ƶ}_{k}\right|}b_{i}^{\left(k\right)}r_{i}^{m}>w_{m}^{\left(k\right)},\\ \; & \forall m,\\ \Delta_{1}>0, & \mathrm{otherwise}, \end{array}\right.$$

where $C_{1}$ and $C_{2}$ are empirically selected constants and $\Delta_{1}>0$ is a constant with very high value. The power consumption of formation $b_{k}$ is $P\left(b_{k}\right)=\sum_{i=1}^{\left|{Ƶ}_{k}\right|}b_{i}^{\left(k\right)}p_{i}$. Function $\bar{\mathrm{sig}}\left(\cdot \right)=L-\frac{L}{1+\exp^{-K(x-x_{0})}}$ is the reflection of the sigmoid function with respect to the vertical axis through the inflection point $x=x_{0}$. The parameters of the reflected sigmoid function are *L*, the maximum value, *K*, the gain and $x_{0}$, the inflection point. By giving the inflection point a value equal to $0.5\;r_{i}^{m}$, the inclusion of this reflected sigmoid function tends to grow singleton terms that describe states where edge servers are under-utilised (i.e., when they serve less than 50% of their nominal workload capacity), close to the maximum value (undesired system state). The intuition behind this design is that the singleton terms express the goal of each site individually for lower energy consumption. Each site strives to reduce its consumption as much as possible, which in turn will drive its singleton term to lower values. At the same time, the term $d\cdot a_{k}$ tends to drive the system towards a solution which keeps the total additional delay, induced by the workload redirections, as low as possible; *d* stands for the single hop network delay in ms while $a_{k}$ corresponds to the ingress workload (i.e., how much additional workload the edge site $s_{k}$ will accommodate, compared to the original).

**Figure 3:**
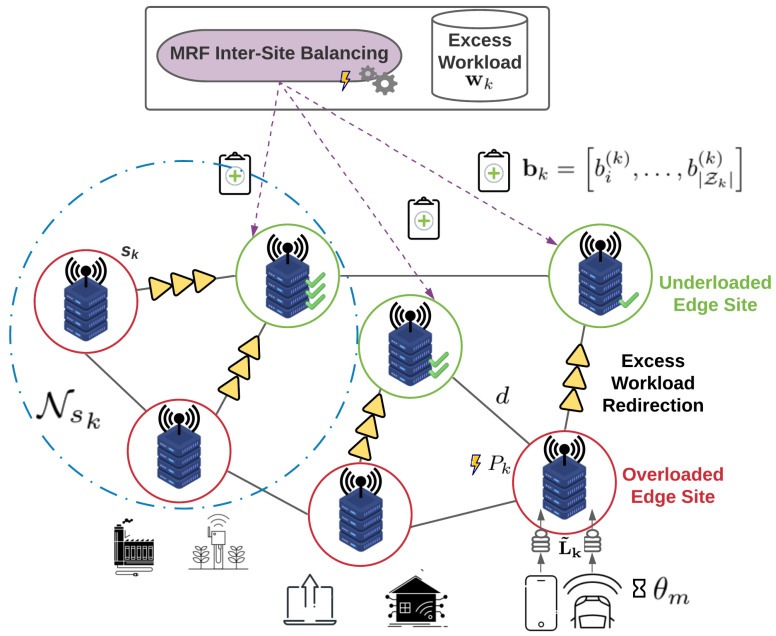
MRF inter-site load redistribution overview (Stage 2).

The doubleton terms are defined as follows:

(12) $$V_{\left\{s_{k},s_{g}\right\}}^{\left(2\right)}\left(x_{k},x_{g}\right)=\left\{\begin{array}{cc} C_{3}w_{k}\cdot w_{g}+C_{4}P\left(b_{g}\right)\left[1+\sum_{m}\bar{\mathrm{sig}}\left(w_{m}^{\left(g\right)}\right)\right], & \mathrm{if}\;\exists \;b_{g}\\ \; & \sum_{i=1}^{\left|{Ƶ}_{k}\right|}b_{i}^{\left(g\right)}r_{i}^{m}>w_{m}^{\left(g\right)},\\ \; & \forall m\\ \Delta_{2}>0, & \mathrm{otherwise}, \end{array}\right.$$

where $C_{3}$ and $C_{4}$ are empirically selected constants and $\Delta_{2}>0$ is again a constant with very high value. The intuition behind the design of the doubleton terms is that, as far as the interactions of the neighboring sites are concerned, ideally we want to drive the system to states where neighboring sites exchange the remaining workload so that it is concentrated in specific sites, thus avoiding having to maintain multiple active sites for a small value of excess workload. It is also important to point out that the MRF activates servers with the appropriate VM flavors as described in Section 3.3. This way, the excess workload is served while respecting the QoS requirement of the maximum acceptable response time. An overview of the MRF-based load redistribution process is depicted in Figure 3.

Each site seeks to minimize its contribution to the cumulative potential function by minimizing its local neighborhood potential function comprised of the sum of its singleton and doubleton (pairwise) potentials with its one-hop neighbors. The state of each site depends only on the states and the information of its neighbors. Gibbs sampling [42] can be applied by each site individually, reaching global optima through local sampling. Cumulatively, this distributed sampling converges to global optimizers of the system. This approach has a very low computational overhead, O(n), with *n* being the number of sites, while reaching asymptotically the global optimal resource allocation solutions, frequently yielding the optimal ones. Furthermore, the signaling overhead is rather small, since each site $s_{k}$ is only required to exchange system state information locally with its one-hop neighbors only.

**Figure 4:**
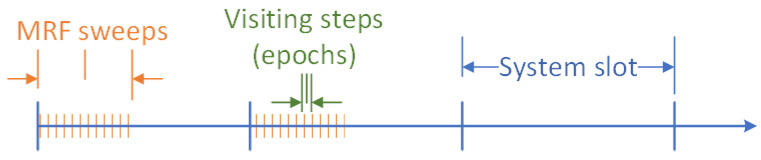
Relation of system slots, sweeps and update epochs.

The sequential Gibbs sampling method proceeds as follows. Consider a logarithmic annealing schedule of the form $T\left(w\right)=\frac{c_{0}}{\ln(1+w)}$, where $c_{0}$ is a constant (equal to 2 in our experiments) and T(w) is called the “temperature” of the *w*-th annealing step. In addition, consider a sequential visiting scheme of all sites, where at each epoch *t* (mini-slot in a sweep) within a step *w*, only one site updates its value (Figure 4 depicts the relations of the system slots, sweeps and update epochs). Starting with an arbitrary initial configuration X(w=0), at epoch *t* of *w*, let ω=X(t) and denote by $\omega^{x_{k}}$ the configuration that has value $x_{k}$ at site $s_{k}$ and agrees with ω everywhere else. The update (decision to transition to a new state) at site $s_{k}$ takes place according to the distribution:

(13) $$P\left(X_{k}\left(t\right)=x_{k}|X_{g}\left(t\right)=x_{g},g\neq k\right)=\frac{\exp(-\frac{1}{T(w)}\sum_{C:s_{k}\in C}V_{C}\left(\omega^{x_{i}}\right))}{\sum_{x_{k}\in \Lambda }\exp(-\frac{1}{T(w)}\sum_{C:s_{k}\in C}V_{C}\left(\omega^{x_{s}}\right))},$$

where *C* is the set of the cliques formed by the sites (here only one-clique and two-cliques are formed in the graph). With probability determined by (13), a site $s_{k}$ will choose $x_{k}$ as its state in sweep w+1. The site states are updated sequentially within a sweep *w*. The annealing schedule represents a decreasing rate of system temperature T(w), where *w* stands for the index of the *w*-th sweep (i.e., the system temperature is updated at the end of each sweep). The *w*-th annealing step is equivalent to the *w*-th sweep, and consists of *n* visiting epochs (denoted by *t* in the above), one for each site. Since sampling begins at high temperatures, where the local characteristics are practically uniform, it permits transitions to higher-potential function configurations, thus avoiding getting trapped in local minima. Thus, in each sweep, the configuration (system state determined by the state of each site) changes. The resulting system states form an inhomogeneous Markov Chain that converges to the uniform distribution on the set of global potential function minimizers. This means that the Markov Chain essentially samples uniformly the whole search space of the problem and thus convergence means that the global optimum has been found. Of course, convergence to the global optimum is guaranteed in infinite time, i.e., the Markov Chain converges in infinite time in the global optimum. In our case, where the number of sweeps is finite, the obtained optimum is in principle suboptimal, but expected to be very close to the global optimum. As shown later, the system indeed exhibits good convergence behavior even for employing a finite number of sweeps.

**Figure 5:**
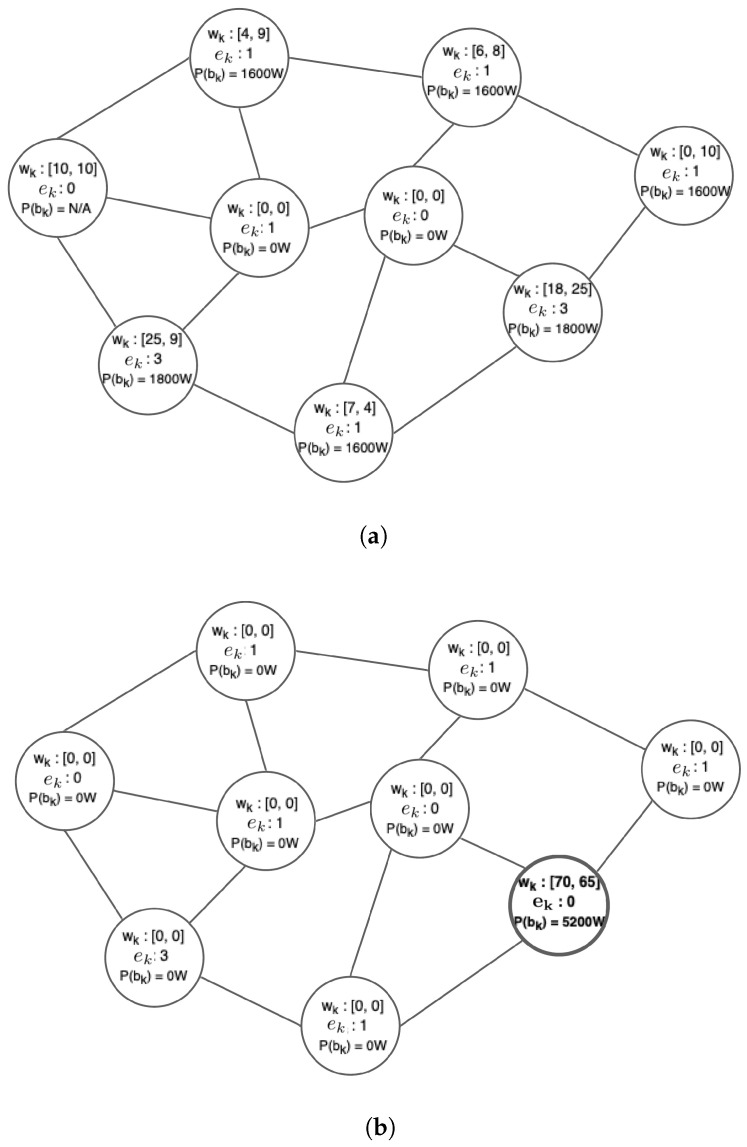
Workload redistribution example: starting and final states. (**a**) starting state; (**b**) final state.

Figure 5 showcases an example of the effect of the MRF-based excess workload redistribution, for two applications in an Edge infrastructure of nine sites, by comparing the starting and final system state (after a finite number of sweeps) where the MRF has converged. As the starting formation for each site, the set of edge servers with the minimum number of allocated resources is selected in order to serve the excess workload locally. It can be observed that, in the final state, the MRF yields a rather desired solution where it has redistributed all the excess requests, $w_{k}$, to a single site, thus minimizing the associated energy consumption of the topology, while serving properly the remaining requests, within the capacity bounds imposed in each site. Specifically, Table 2 shows the selected VM formation for the particular site, with three activated servers.

We observe that this site formation fits to accommodate the workload. The total power consumption, $P(b_{k})$, is 5200 W, which is around half of the 10,000 W power consumption of the initial site formations selected, had the excess workload been executed locally. The number of available servers per site $e_{k}$, is also depicted. In addition, local execution would lead to some requests being rejected, as there is one site that has no available servers to accommodate its excess workload. Consequently, the MRF based mechanism emerges as rather effective in increasing the energy efficiency of the whole approach.

### 4.3. ENERDGE Core Algorithm

In this subsection, the core algorithm of a full ENERDGE deployment in an edge infrastructure, as well as its importance, is summarized. At first, the required datasets are produced and the VM flavor design procedure is performed offline. Then, as shown in Algorithm 1, the initial optimization and the distributed resource allocation for each site of the edge infrastructure take place, as explained in the previous sections.

During this online phase, in Stage 1, the density of users and devices is predicted using the *n*-MMC method. The incoming workload at each site of the infrastructure is estimated for the current system slot. The resource allocation optimization produces an initial solution subject to QoS and energy constraints for a given predicted workload at each site. Then, in Stage 2, for each site, the excess predicted workload or workload that cannot be served, along with the available resources, are computed. The excess workload is redistributed between the extra servers activated in under-loaded sites, according to the MRF solution, achieving the minimization of the energy consumption for the edge infrastructure.

**Algorithm 1:** ENERDGE core algorithm. **Data**:Trajectory Dataset **Result**:Optimal VM placement in Edge Infrastructure
 
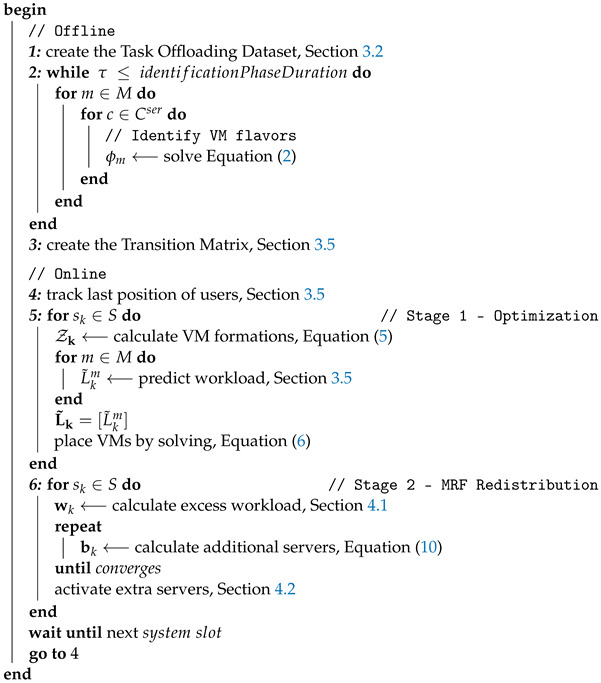



Precisely estimating the needed resources for an edge infrastructure can be a great challenge, as users’ behavior and thus offloaded workload can vary in different conditions. In this context, the proposed two-stage solution brings significant benefits in the problem at hand. In particular, the offline analysis helps at creating a throughput (or offloading request rate) heatmap and a user density heatmap for the infrastructure, based on experienced network conditions and user density patterns. Then, the first stage, which is based on the outcome of this analysis, gives a first, rough resource allocation solution. However, the behavior of the users or the network conditions cannot always be predicted; in this case, this first-stage planning will fail, which may cause severe impact in the perceived QoS. Thus, the second stage helps to refine the first solution and to account for the inequalities between the predicted requirements and the actual needs. This can further guarantee the QoS requirements of the applications, while also minimizing the energy consumption at the Edge infrastructure.

## 5. Performance Evaluation

In this section, the performance of the proposed resource allocation and excess load redistribution mechanism is presented via modeling and simulation. The results illustrate the success of our approach in minimizing the energy consumption while guaranteeing the stability of the application’s QoS (i.e., response time) within an acceptable margin. We highlight the optimization of the resource allocation in terms of the power consumption of the activated edge servers and the VM flavors used to serve the incoming workload. The benchmarking is conducted using CloudSim Plus [43], a Java-based simulator suitable for Edge and Cloud environment experimentation. Then, a comparison with one well-established study in the literature and additionally with a more recent one follows.

### 5.1. Smart Museum Experiment Setting

To demonstrate the operation of an ENERDGE real-world application, we emulate the environment of a smart museum. The museum accommodates different categories of interactive exhibits, and it is equipped with a large number of IoT sensors and edge devices with heterogeneous computational capabilities. Furthermore, the dynamic network conditions are modeled by the dynamic behavior and density of the users. In particular, our physical infrastructure consists of nine interconnected interactive exhibits-sites resembling to a smart museum floor plan. Each site hosts an edge data center which includes three edge servers. The applications deployed in the museum are classified in two categories with different characteristics and requirements:

**Interactive Exhibit Apps:** On the one hand, we consider the museum leveraging Augmented Reality (AR) and Virtual Reality (VR) settings to provide rich and detailed access to artwork and artifacts, bring life to works of art and allow visitors to engage in adaptable visual guided tours by using their mobile devices. In order to achieve the high QoS requirements of these types of applications, mobile devices can offload some workload by sharing video decoding tasks to the more powerful edge devices. User density is highly dynamic in these applications, as visitors move from one exhibit to the other.

**Sensor Monitoring Apps:** On the other hand, IoT is making it possible to deploy low-cost, automated monitoring of collections and museum facilities, e.g., static sensors for temperature, humidity, counting number of visitors, etc. Such applications exhibit low delay requirements, i.e., the processing can be performed in a delay tolerable manner, sending data and information after a completion of an activity. However, they produce numerous requests to the edge servers.

We assume one application of the Interactive Exhibit type, denoted as *App1*, and one of the Sensor Monitoring type, denoted as *App2*, co-hosted in each site. This means that VMs of both application types are able to run simultaneously in the edge servers, receiving offloading requests from their counterparts in the visitors’ mobile devices and the IoT sensors, respectively. For demonstration purposes, we also assume that both apps are based on image recognition processes, thus their acceptable response time (QoS) is set at 3 s, which lies within the margins of a typical image recognition service time [44] and provides a satisfying Edge Computing AR application experience to the user [45]. As the design of our framework and modeling of the applications are independent of the level of the applications QoS requirements, applications that require lower (or higher) response times are naturally supported. Following the modeling approach explained in Section 3.3, we identify the VM flavors shown in Table 3, tuned towards achieving the above QoS requirement. It should be noted here that *App1* requests need considerably heavier computations to achieve this response time than the ones of *App2*. This limits the maximum number of requests of the application *App1* to one third of those that can be served by the *App2* for equally sized VMs. Sixty visitors are assumed to roam the museum at each given time, offloading requests for App1, while twenty static sensors are assumed to be deployed, producing offloading requests for App2 at a much higher rate. The system slot is arbitrarily set at 30 s and the experiments last for a period of 1 h, or 120 system slots. The simulation code alongside any related dataset used in this section is publicly available (https://github.com/maravger/netmode-cloudsim, accessed on 30 November 2021).

### 5.2. Resource Allocation Evaluation

In this subsection, we present the evaluation of the resource allocation algorithm. At first, the impact of the selected user density prediction method is assessed and then a summary of the core optimization results for Stages 1 and 2 are provided. Finally, a comparison of the whole mechanism with two works on the field is demonstrated.

#### 5.2.1. User Density Prediction Impact

**Figure 6:**
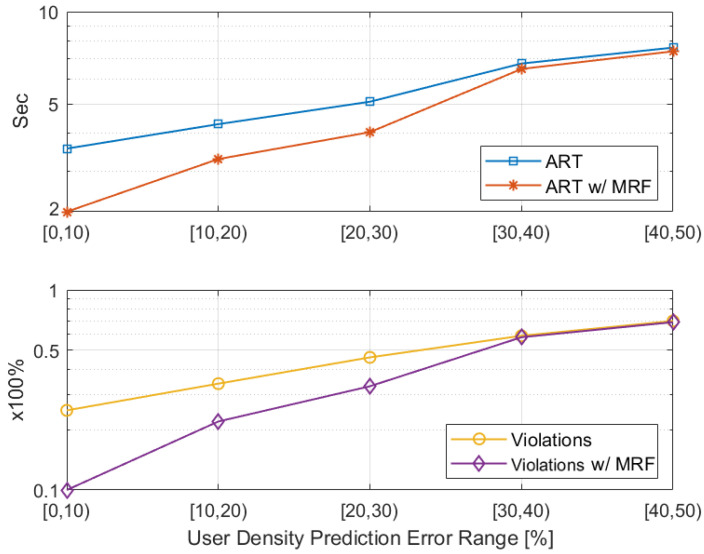
ART & QoS violations sensitivity to prediction error.

As described in Section 4.3, predicting the visitors’ positions in the next system slot is the first step of optimizing the allocation of the edge resources in each site. This provides an estimation on the projected workload. To quantify the impact of the user density prediction accuracy, a sensitivity analysis is performed as illustrated in Figure 6; this assesses the impact of the prediction error on satisfying the required application QoS, both in terms of the average response time (ART) per request and the percentage of the violations occurred in respecting the QoS. A logarithmic scale is used to better visualize both impacts in a combined fashion.

We opted for showcasing the impact analysis at the end of both Stages of the resource allocation mechanism, separately, so as to highlight the significant effect the MRF-based workload redistribution has on alleviating the disruptions caused by the prediction error. The results are collected from running the simulation for 10,000 system slots, for various topologies, and averaging the stats in batches of 10. Thus, the *x*-axis of Figure 6 represents the range of the prediction error. The dataset used is again the Melbourne Museum one [39].

Underestimating the real incoming workload leads to under-provisioning of resources and subsequently to slight degradation of the response time. In detail, we notice that both the ART and the violations grow almost linearly with the prediction error. It is also clear that the application of the MRF-based redistribution in each system slot has a great impact on respecting the QoS requirements, with the redirection of the excess projected workload from overutilised sites to underutilised ones. Specifically, when the MRF is applied, the ART lies around 2 s and the QoS violations do not exceed 10% of the offloaded requests, when the prediction error is less than 10%. The ART grows to around 3 s, which is still acceptable for both applications, and the violations to 20%, when the error is less than 20%. Beyond the point of a 30% prediction error, we notice that our solution’s results converge to those of the naive one, as the extra unpredicted workload puts excessive strain on the mechanism. However, this should not be a problem, as selecting an appropriate prediction mechanism, like the *n*-MMC used here and in other comparable works, e.g., [46], leads to an average prediction accuracy of 70–95%.

#### 5.2.2. Stage 1 Evaluation—Response to Dynamic Network Conditions

**Figure 7:**
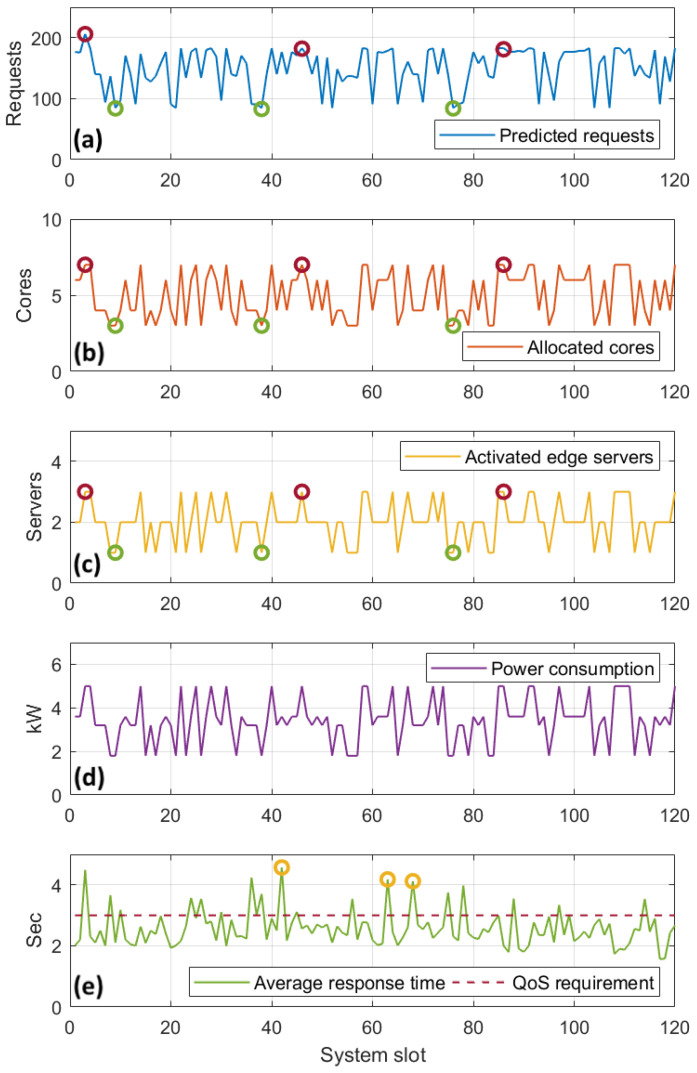
Dynamic resource allocation: Allocated cores (**b**), number of activated edge servers (**c**), power consumption (**d**) and ART (**e**) as a response to the number of predicted requests (**a**), for a single site.

In this subsection, we closely examine how the resource allocation optimization reacts to the dynamic workload demands caused by the visitors’ dynamic density on each site, in terms of edge servers activated and the VMs placed in them. Figure 7 showcases the scalability of the proposed technique, as a response to the population of the visitors’ devices and the fluctuations in the sensors’ offloading rate. We present the behavior of a single site, which is equipped with three servers of four cores each, and this acts as a baseline for the rest of the evaluation. With regard to power consumption, for demonstration purposes, we assume that the average maximum power consumption of an edge server is 2000 W, in accordance with [47].

Figure 7a shows the predicted workload per system slot, as calculated in the previous step, while Figure 7b–d demonstrate how the resource optimizer adapts to the fluctuations. In particular, they depict how the optimizer selects the appropriate topology in terms of number of active edge servers and their allocated cores, in order to meet the demands for the selected site. For instance, when the predicted requests are high, e.g., at system slots {3,46,86}, with {206,182,181} predicted requests respectively for both applications (red-colored marks), our optimization results in three activated edge servers and seven cores allocated among them. On the other hand, when the incoming request prediction is considerably lower, as in system slots {9,38,76}, with {84,83,84} predicted requests, respectively (green-colored marks), only one server with three allocated cores is activated. The results corroborate the total power consumption, as shown in Figure 7d.

Exploring further, we demonstrate an example regarding the specific VM formations selected for the above activated servers, at system slot 3. The total of 206 predicted requests consisted of 17 requests for *App1* and 189 requests for *App2*. Table 4 shows the selected VM formation for the three activated servers for this system slot. We see that this VM formation fits to accommodate the predicted workload. The site’s power consumption, in this slot, is 5000 W.

While our approach adapts very well against the various predicted incoming workloads in terms of allocated resources, satisfying the QoS for these applications is challenging. This is due to the fact that the VM topology to serve these requests is selected based on the predicted workload which is potentially fallacious, as explained in the previous subsection, and this leads to violations in the QoS. For instance, as shown in Figure 7e, in system slots {42,63,68} (yellow-colored marks), the average response time for both applications was slightly above 4 s, or approximately 35% larger than the reference value, set at 3 s. This is an indication of under-provisioning due to incoming workload underestimation. Violations like this took place 17 times in this site, or 14% in a total of 120 system slots. We consider this to be an acceptable margin of error for the satisfaction of the perceived QoS. Finally, it should be pointed out that, for this experimentation, the average service completion time mainly affected the measured response time. The average transmission time is negligible, due to the use of the IEEE 802.11ac standard, which provides high throughput for requests of application types used in this experiment.

#### 5.2.3. Stage 2 Evaluation—MRF-Based Excess Workload Redistribution Analysis

**Figure 8:**
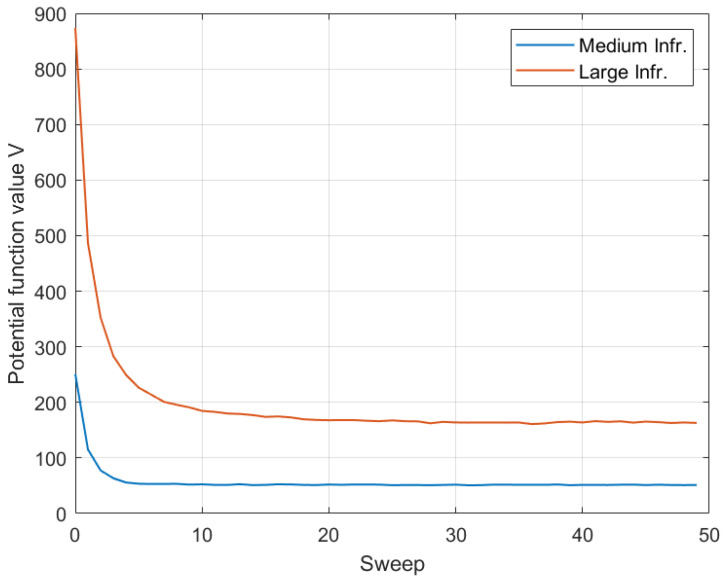
MRF-based workload redistribution convergence.

In this subsection, initially we demonstrate the convergence behavior of the MRF approach for a standard (medium-size) and a larger topology. Figure 8 demonstrates the variation of the cumulative potential function of the MRF (Equation (10)) for a complete set of sweeps corresponding to an execution of the MRF in the beginning of a system slot. The results of this evaluation have been averaged over 100 different topologies, both for a 9-site (Medium) and a 36-site (Large) Edge infrastructure.

It is observed that the Gibbs sampler converges rather quickly, and it succeeds in reducing the variability of the potential value rapidly. This is because the sampler is a uniform global optimizer of the state space, and it is able to identify the local neighborhood of desired solutions relatively fast, within the first five sweeps, and then fine-tune the search, eventually selecting one solution among the global minimizers of the potential function. As expected, the larger topology exhibits greater variability of the cumulative potential function in the first sweeps (due to a larger state space), but eventually convergence is smooth and within the maximum number of designated sweep iterations (here employing a maximum of 50 sweeps).

**Figure 9:**
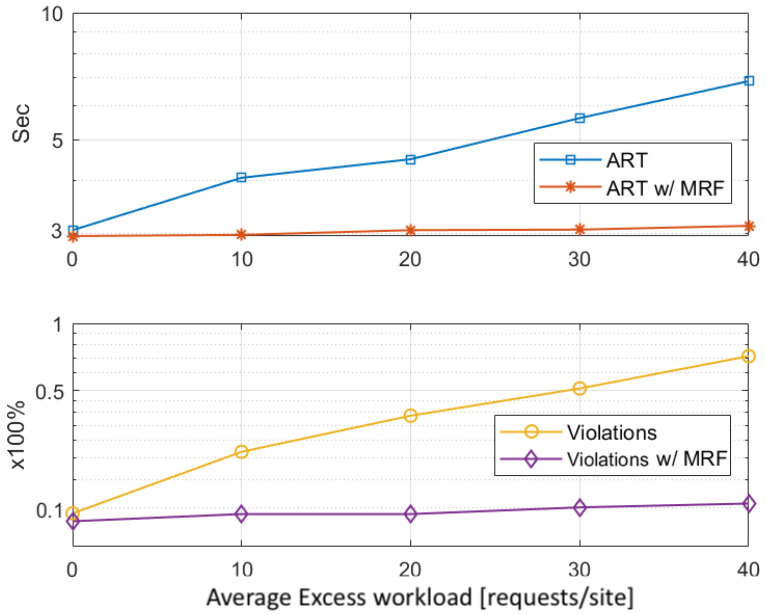
MRF QoS improvements for various excess workloads in overloaded sites.

To evaluate the efficiency of this second stage of our mechanism, as discussed in Section 4.2, we identify two cases of excess workload at the end of the first stage. Regarding the workload coming from overloaded sites, Figure 9 depicts the improvement in the QoS satisfaction that comes with the application of the MRF-based redistribution (in a logarithmic scale). We observe that, while both the ART and the violations metrics grow almost linearly with the average excess workload (in requests per site), by applying the MRF-based redistribution, our mechanism achieves to provide better QoS guarantees (i.e., ART ≈ 3 s and violations ≈10%). This comes as a natural result, since the overloaded sites are alleviated from the excess workload, which is redistributed throughout the infrastructure.

**Figure 10:**
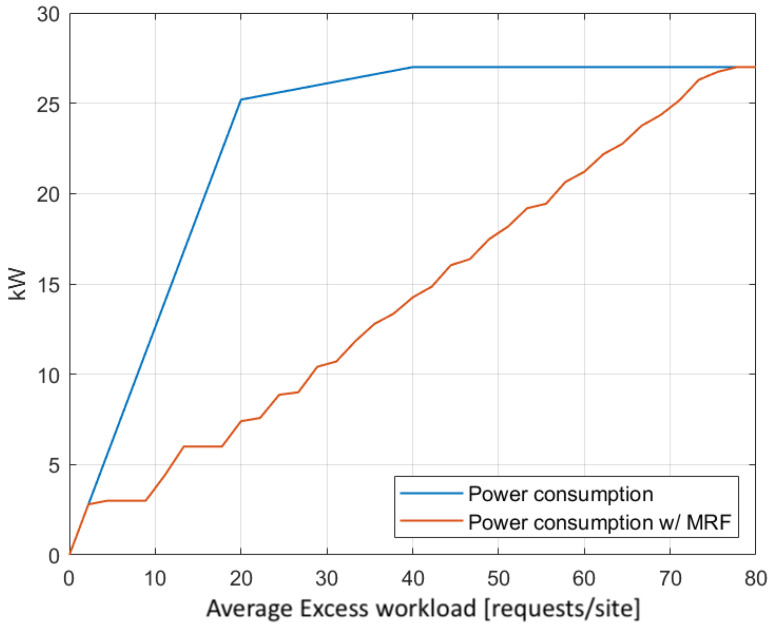
MRF energy savings for various excess workloads in underloaded sites.

On the other hand, regarding the underloaded sites, Figure 10 demonstrates the effect of the MRF-based excess workload redistribution on the total energy consumption of the infrastructure, by comparing it to the case where no redistribution of any kind takes place. During the latter, as the average excess workload increases, the power consumption increases radically, as underloaded edge servers are activated in each site in order to accommodate the low volume of excess requests locally. From that point on, power consumption increases moderately, as larger VMs are provisioned to meet the increasing workload demands, until the point where all the resources are allocated in each site and the maximum power consumption of the infrastructure is reached. In contrast, when the MRF-based redistribution is employed, power consumption adjustment is more fine-grained, as only the minimum combination of activated servers and installed VMs flavors are deployed in each case.

**Figure 11:**
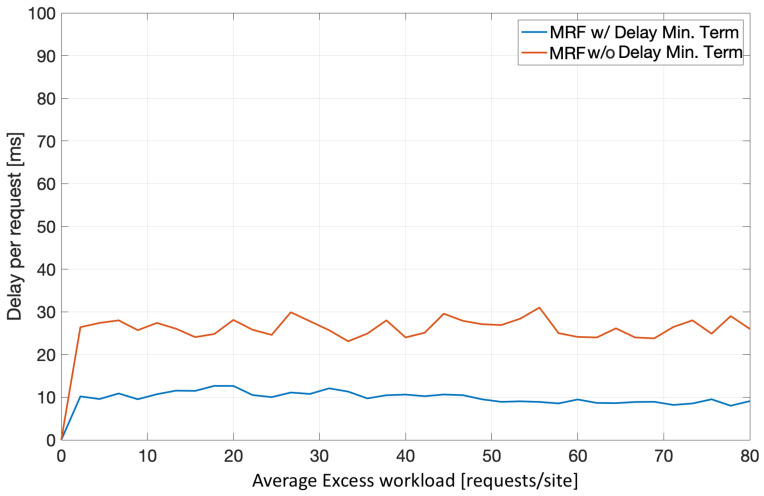
MRF workload redistribution-induced delay minimizing for various excess workloads.

Finally, Figure 11 illustrates the impact of the delay minimizing term in the MRF-based workload redistribution. It is clear that the delay-related term in the MRF-based solution minimizes the redirection-induced overhead per request (≈10 ms average), when compared to an MRF-solution without it (≈26 ms average), in a medium sized edge infrastructure. It should also be noted that the inclusion of this term has an impact on the average additional delay being far more stable throughout the average excess workload increase.

### 5.3. Two-Stage Approach Comparison

In the following, we present a comparative evaluation of the overall resource allocation of ENERDGE with two works, presented in [11,12], respectively. This comparison highlights the ability of our two-stage approach to minimize energy consumption in the edge infrastructure, while guaranteeing a certain level of QoS. Similar to our study, Jia et al. in [11] present a setting of dispersed and interconnected clusters of computers, namely *cloudlets*, which form a wireless metropolitan area network. Contrary to ENERDGE, each cloudlet has a static VM provisioning method to serve offloaded requests. This study focuses on identifying over-utilized cloudlets and redirecting part of their incoming workload to under-utilized ones in order to achieve better resource utilization. On the other hand, in [12], Zhang et al. present a system of multiple distributed and interconnected intelligent edge servers (*IESs*), located in an urban region. Again, in this work, the computing resources are statically allocated to serve the offloaded requests coming from mobile devices and the focus is placed on balancing this load between the IESs through workload redistribution, using a novel game theoretic perspective together with a state-based distributed learning algorithm. For both works, instead of having an estimation of the incoming workload, it is considered known for each cloudlet/IES and for each system slot. In addition, the offloaded workload served at each cloudlet/IES is bounded by its service rate capabilities, while the rest of it is rejected and redirected back to the mobile device for local execution.

In order to highlight the importance of dynamic resource allocation towards simultaneously guaranteeing the QoS requirements and minimizing energy consumption, we compare our method with two differently oriented resource provisioning settings of [11,12], resulting in two sets of experiments. For the first one (Experiment A), all three works attempt to minimize energy consumption, while in the second one (Experiment B), the effort is put on satisfying the QoS constraints. To make the comparison fair, we simulated the exact same nine-site edge infrastructure, described in Section 5.1, for all three methods. The generated workload traffic is the same for all methods as well.

**Figure 12:**
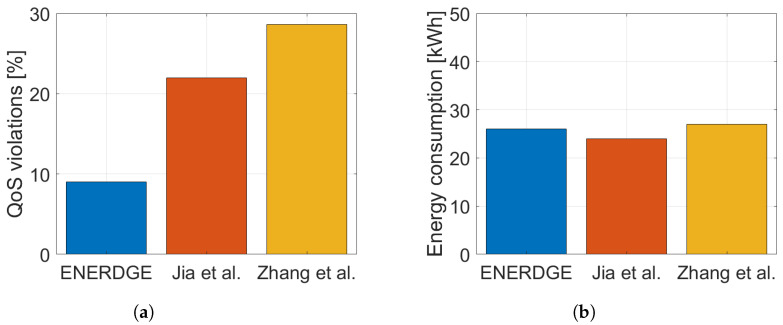
QoS violations and energy consumption during Experiment A. (**a**) QoS violations; (**b**) energy consumption.

Regarding Experiment A, we chose a frugal static resource allocation for both [11,12], so that they would approximately match the total energy consumption of ENERDGE (Figure 12b). QoS violations were calculated for all methods based on the SLA threshold for the response time of the offloaded requests, set at 3 s, as in Section 5.2.2. In one hour of experimentation, the ENERDGE sites reported 207 violations, or 9% of the offloaded requests, compared to the 470 violations or 22% of the requests in [11] and 660 violations or 29% of the requests in [12], as shown in Figure 12a.

**Figure 13:**
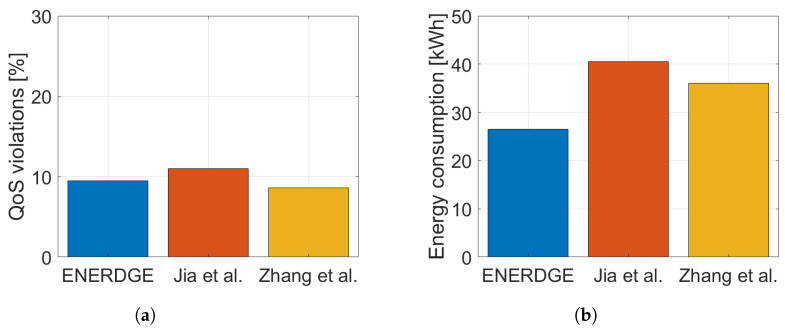
QoS violations and energy consumption during Experiment B. (**a**) QoS violations; (**b**) energy consumption.

On the contrary, in Experiment B, resource-abundant static allocations were selected for the other two works, in order to match the QoS satisfaction of ENERDGE (Figure 13a). In this case, as shown in Figure 13b, energy consumption for one hour in [11] was roughly 41 kWh and in [12] 36 kWh, or more than 54% and 35% bigger, respectively, when compared to the 26.5 kWh of our method. In addition to the previous results, it is clear that even a static resource provisioning method enhanced with workload redirection mechanisms is incapable of finding a balance between QoS satisfaction and infrastructure energy consumption minimization, the way ENERDGE does.

**Figure 14:**
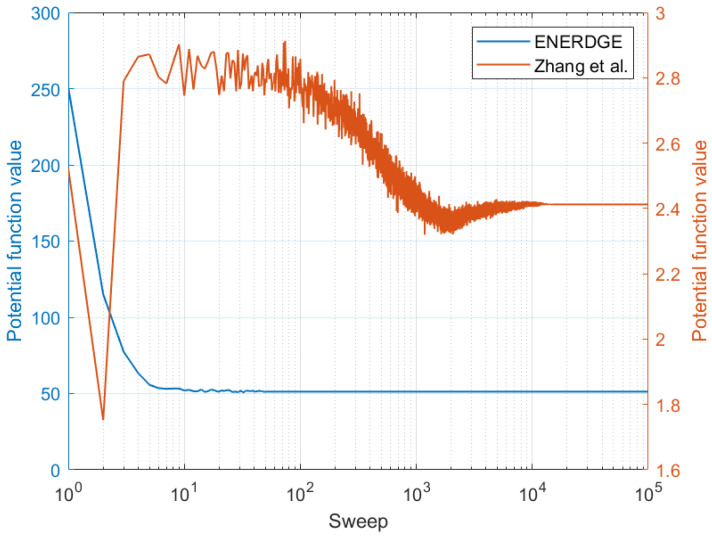
Comparison of the workload redistribution convergence.

Finally, as the work in [12] incorporates a game theoretic solution and a decentralised learning algorithm, an opportunity arises for comparing the convergence behavior of the MRF solution with it. In Figure 14, the potential function values for both solutions are illustrated in a logarithmic scale, with respect to each algorithm’s iterations, after averaging over 1000 executions of a random 9-site infrastructure and similar offloaded workload for both. The results reveal that the proposed MRF solution converges rapidly compared to the solution proposed in [12], which also has a direct effect on our mean execution times being significantly lower.

## 6. Conclusions

This article introduced the ENERDGE framework that addresses jointly the full task offloading and resource allocation problems in a multi-site setting. We proposed a holistic energy-aware resource optimization approach, based on the design of the VM flavors complemented with an innovative load redistribution technique based on MRFs, with the penultimate goal to minimize the total energy consumption without sacrificing the QoS in terms of latency. To minimize the inverse impact of the dynamic presence of users, ENERDGE considers the dynamic wireless conditions of the access network and supports a mobility prediction scheme to better guide the allocation solution during task offloading. Numerical results showed that the prediction mechanism accurately predicts the mobile behavior of the users, while the ENERDGE resource optimizer outperforms two well-established load balancing techniques in terms of both latency and energy consumption. Finally, we have shown that the MRF scheme converges rapidly to minimum energy solutions, thus allowing further energy optimizations in an efficient manner.

Our future work will concentrate on the interplay between the Edge and Cloud. As IoT and cellular device volumes continue to increase, a collaboration between the Edge and Cloud infrastructures may constitute a viable solution for large-scale deployment scenarios. Furthermore, integrating machine learning techniques in our user density prediction approach will enable addressing errors in the predictions of the dynamically estimated values of the position and number of end-user devices.

## Figures and Tables

**Table 1:** Summary of the Key Notations.

Symbol	Interpretation
$s_{k}$	Site *k*
*S*	Set of sites, n=|S| sites in total
*M*	Number of applications
$\theta_{m}$	Acceptable response time for App. *m*
$\phi_{m}$	VM flavor of application *m*
$c_{m}$	Cores requested by VM flavor $\phi_{m}$
$\mu_{m}$	Throughput guaranteed by VM flavor $\phi_{m}$
$Ser_{cpu}$	Server’s CPU capacity
$P_{ser}$	Server’s power consumption
$P_{max}$	Server’s max. power consumption
$P(\phi_{m})$	Power consumption of VM flavor $\phi_{m}$
$z_{i}$	A feasible VM formation
${Ƶ}_{k}$	Set of feasible VM formations at site $s_{k}$
*N*	Size of $z_{i}$ VM formation
$C_{k}^{ser}$	Servers’ CPU cores threshold at site $s_{k}$
$P_{A}$	Edge infrastructure’s power consumption
$P_{k}$	Power consumption of site $s_{k}$
$f_{i}$	Number of servers with $z_{i}$ VM formation
$E_{k}$	Number of available servers in site $s_{k}$
$p_{i}$	Power consumption of VM formation $z_{i}$
$r_{i}^{m}$	Max. workload served by VM formation $z_{i}$
${\tilde{L}}_{k}=\left[{\tilde{L}}_{k}^{m}\right]$	Predicted workload for site $s_{k}$
$N_{s_{k}}$	Neighborhood of site $s_{k}$
$w_{k}=\left[w_{m}^{\left(k\right)}\right]$	Excess workload for App. *m* at site $s_{k}$
$b_{k}=\left[b_{i}^{\left(k\right)}\right]$	Number of servers of type *i* at site $s_{k}$
$P(b_{k})$	Power consumption of $b_{k}$
$X_{k}={\left\{W_{k},B_{k}\right\}}_{k=1}^{n}$	Random field
V(ω)	MRF potential function
$C_{1},C_{2},C_{3},\Delta_{1},\Delta_{2}$	Properly selected MRF constants
*L*, *K*, $x_{0}$	Parameters of reflected sigmoid function
*t*	Visiting epoch of MRF
*w*	MRF sweep index
T(w)	MRF temperature at sweep *w*

**Table 2:** VM formations selected by the MRF mechanism.

Server ($b_{k}$)	*App1* VMs	*App2* VMs
1	1 × medium	1 × small
2	1 × medium	1 × small
3	1 × medium	-
**Site Workload** **Capacity **$\left(\sum_{i=1}^{\left|{Ƶ}_{k}\right|}b_{i}^{\left(k\right)}r_{i}^{m}\right)$	81	82

**Table 3:** Identified VM Flavors.

Flavor	Small		Medium		Large	
	*App1*	*App2*	*App1*	*App2*	*App1*	*App2*
Cores	1	1	2	2	4	4
QoS (s)	3	3	3	3	3	3
Maximum Requests/Slot	11	38	27	82	59	173

**Table 4:** VM formations in Slot 3.

Server	App1 VMs	App2 VMs	Allocated Cores
1	1 × small	1 × medium	3
2	1 × small	1 × medium	3
3	-	1 × small	1
Site Workload Capacity	22	202	

## Data Availability

Publicly available datasets were generated in this study. These data can be found here: https://github.com/maravger/netmode-cloudsim/blob/master/task_offloading_ds_verbose.xlsx, accessed on 30 November 2021.

## Appendix A

Assume a finite set *S*, |S|=n, with elements s∈S referred to as sites or nodes. These correspond to access points of the considered infrastructure. Every site *s* is associated with a random variable $X_{s}$ that expresses its state. In our case, the state of each site will depend on the excess workload and number of assigned servers. Let the phase space Λ be the set of possible states of each s∈S, i.e., $X_{s}$ takes a value $x_{s}\in \Lambda$. The collection $X=\{X_{s},\;\forall s\in S\}$ of random variables with values in Λ consists of a Random Field (RF) on *S* with phases in Λ. A configuration $\omega =\{x_{s}:x_{s}\in \Lambda ,\;\forall s\in S\}$ corresponds to one of all possible states of the system and the product space $\Lambda^{n}$, $\omega \in \Lambda^{n}$ denotes the configuration space. A neighborhood system on *S* is defined as a family $N={\left\{N_{s}\right\}}_{s\in S}$ of subsets $N_{s}\subset S$, such that for every s∈S, $s\notin N_{s}$ and $r\in N_{s}$ if and only if $s\in N_{r}$. $N_{s}$ is called the neighborhood of site (node) *s*. The RF *X* is called a Markov Random Field (MRF) with respect to N, if for every site s∈S,

(A1) $$\begin{array}{c} P\left(X_{s}=x_{s}|X_{r}=x_{r},r\neq s\right)=P\left(X_{s}=x_{s}|X_{r}=x_{r},r\in N_{s}\right). \end{array}$$

A RF *X* is called a Gibbs Random Field (GRF) if it satisfies:

(A2) $$P\left(X=\omega \right)=\frac{1}{Z}e^{-\frac{U(\omega )}{T}},$$

where $Z:=\sum_{\omega \in \Lambda^{n}}e^{-\frac{U(\omega )}{T}}$ is the partition function and *T* is the temperature of the system. U(ω) is called the potential function and represents a quantitative metric of the current state of the configuration ω. The potential function is not unique. A very useful class of potential functions, which we will employ in our approach, is one in which U(ω) is decomposed into a sum of clique (maximally connected subgraph) potential functions, as $U\left(\omega \right)=\sum_{c\in C}V_{c}\left(\omega \right)$, where C is the set of the cliques formed by the sites and each clique potential $V_{c}$ depends only on the states of the cliques formed in the underlying system graph. The Hammersley–Clifford theorem [41] asserts that a GRF with distribution $P\left(X=\omega \right)=\frac{1}{Z}e^{-\frac{U(\omega )}{T}}$ and potential function expressed in terms of clique potentials leads to an MRF with conditional probabilities $P\left(X_{s}=x_{s}|X_{r}=x_{r},r\neq s\right)=P\left(X_{s}=x_{s}|X_{r}=x_{r},r\in N_{s}\right)$ and vice versa. This property is also employed for the design of the potential function and the implementation of distributed decision-making via Gibbs sampling.

## References

[1] Jeon Y., Baek H., Pack S. Mobility-aware optimal task offloading in distributed edge computing. Proceedings of the 2021 International Conference on Information Networking (ICOIN).

[2] Bebortta S., Senapati D., Panigrahi C.R., Pati B. (2021). An adaptive performance modeling framework for QoS-aware offloading in MEC-based IIoT systems. IEEE Internet Things J..

[3] Sahni Y., Cao J., Zhang S., Yang L. (2017). Edge mesh: A new paradigm to enable distributed intelligence in internet of things. IEEE Access.

[4] Li S., Zhang N., Lin S., Kong L., Katangur A., Khan M.K., Ni M., Zhu G. (2018). Joint admission control and resource allocation in edge computing for internet of things. IEEE Netw..

[5] Thai M.T., Lin Y.D., Lai Y.C., Chien H.T. (2019). Workload and capacity optimization for cloud-edge computing systems with vertical and horizontal offloading. IEEE Trans. Netw. Serv. Manag..

[6] Xia X., Chen F., He Q., Grundy J., Abdelrazek M., Jin H. (2020). Online collaborative data caching in edge computing. IEEE Trans. Parallel Distrib. Syst..

[7] Li Y., Wang S. An energy-aware edge server placement algorithm in mobile edge computing. Proceedings of the 2018 IEEE International Conference on Edge Computing (EDGE).

[8] Daraghmeh M., Al Ridhawi I., Aloqaily M., Jararweh Y., Agarwal A. A power management approach to reduce energy consumption for edge computing servers. Proceedings of the 2019 Fourth International Conference on Fog and Mobile Edge Computing (FMEC).

[9] Avgeris M., Spatharakis D., Dechouniotis D., Kalatzis N., Roussaki I., Papavassiliou S. (2019). Where there is fire there is smoke: A scalable edge computing framework for early fire detection. Sensors.

[10] Gambs S., Killijian M.O., del Prado Cortez M.N. Next, place prediction using mobility markov chains. Proceedings of the MPM ’12-First Workshop on Measurement, Privacy, and Mobility.

[11] Jia M., Liang W., Xu Z., Huang M. Cloudlet load balancing in wireless metropolitan area networks. Proceedings of the IEEE INFOCOM 2016-The 35th Annual IEEE International Conference on Computer Communications.

[12] Zhang F., Deng R., Zhao X., Wang M.M. (2021). Load Balancing for Distributed Intelligent Edge Computing: A State-based Game Approach. IEEE Trans. Cogn. Commun. Netw..

[13] Guo J., Song Z., Cui Y., Liu Z., Ji Y. Energy-efficient resource allocation for multi-user mobile edge computing. Proceedings of the GLOBECOM 2017—2017 IEEE Global Communications Conference.

[14] Saeik F., Avgeris M., Spatharakis D., Santi N., Dechouniotis D., Violos J., Leivadeas A., Athanasopoulos N., Mitton N., Papavassiliou S. (2021). Task offloading in Edge and Cloud Computing: A survey on mathematical, artificial intelligence and control theory solutions. Comput. Netw..

[15] Dechouniotis D., Athanasopoulos N., Leivadeas A., Mitton N., Jungers R.M., Papavassiliou S. (2020). Edge Computing Resource Allocation for Dynamic Networks: The DRUID-NET Vision and Perspective. Sensors.

[16] Wang L., Jiao L., Li J., Mühlhäuser M. Online resource allocation for arbitrary user mobility in distributed edge clouds. Proceedings of the ICDCS 2017—The 37th IEEE International Conference on Distributed Computing Systems.

[17] Puliafito C., Mingozzi E., Vallati C., Longo F., Merlino G. Companion fog computing: Supporting things mobility through container migration at the edge. Proceedings of the IEEE SMARTCOMP 2018—The 4th IEEE International Conference on Smart Computing.

[18] Labriji I., Meneghello F., Cecchinato D., Sesia S., Perraud E., Strinati E.C., Rossi M. (2021). Mobility aware and dynamic migration of MEC services for the Internet of Vehicles. IEEE Trans. Netw. Serv. Manag..

[19] Plachy J., Becvar Z., Strinati E.C. Dynamic resource allocation exploiting mobility prediction in mobile edge computing. Proceedings of the IEEE PIMRC 2016—27th IEEE International Symposium on Personal, Indoor and Mobile Radio Communications.

[20] Sun X., Ansari N. (2017). Adaptive avatar handoff in the cloudlet network. IEEE Trans. Cloud Comput..

[21] Shi Y., Chen S., Xu X. (2017). MAGA: A mobility-aware computation offloading decision for distributed mobile cloud computing. IEEE Internet Things J..

[22] Al-Shuwaili A., Simeone O. (2017). Energy-efficient resource allocation for mobile edge computing-based augmented reality applications. IEEE Wirel. Commun. Lett..

[23] Elgendy I.A., Zhang W.Z., Zeng Y., He H., Tian Y.C., Yang Y. (2020). Efficient and secure multi-user multi-task computation offloading for mobile-edge computing in mobile IoT networks. IEEE Trans. Netw. Serv. Manag..

[24] Ren J., Yu G., Cai Y., He Y. (2018). Latency optimization for resource allocation in mobile-edge computation offloading. IEEE Trans. Wirel. Commun..

[25] Farris I., Militano L., Nitti M., Atzori L., Iera A. (2017). MIFaaS: A mobile-IoT-federation-as-a-service model for dynamic cooperation of IoT cloud providers. Future Gener. Comput. Syst..

[26] Sonmez C., Ozgovde A., Ersoy C. (2019). Fuzzy workload orchestration for edge computing. IEEE Trans. Netw. Serv. Manag..

[27] Jia M., Liang W., Xu Z., Huang M., Ma Y. (2018). Qos-aware cloudlet load balancing in wireless metropolitan area networks. IEEE Trans. Cloud Comput..

[28] Leivadeas A., Nilsson Y. T., Elahi A., Keyhanian A., Lambadaris I. Link Adaptation for Fair Coexistence of Wi-Fi and LAA-LTE. Proceedings of the ACM MobiWac 2018—The 16th ACM International Symposium on Mobility Management and Wireless Access.

[29] Erceg V. (2004). IEEE 802.11-03/940r4.

[30] Madwifi Project—Minstrel Algorithm. https://sourceforge.net/p/madwifi/svn/HEAD/tree/madwifi/trunk/ath_rate/minstrel/minstrel.txt.

[31] Tran T.X., Pompili D. (2018). Joint task offloading and resource allocation for multi-server mobile-edge computing networks. IEEE Trans. Veh. Technol..

[32] Leivadeas A., Papagianni C., Papavassiliou S. (2015). Going Green with the Networked Cloud: Methodologies and Assessment. Wiley Quantitative Assessments of Distributed Systems: Methodologies and Techniques.

[33] Cuervo E., Balasubramanian A., Cho D.k., Wolman A., Saroiu S., Chandra R., Bahl P. MAUI: Making smartphones last longer with code offload. Proceedings of the ACM MobiSys 2010—The 8th Annual International Conference on Mobile Systems, Applications, and Services.

[34] Kosta S., Aucinas A., Hui P., Mortier R., Zhang X. Thinkair: Dynamic resource allocation and parallel execution in the cloud for mobile code offloading. Proceedings of the IEEE INFOCOM 2012—The 31st Annual IEEE International Conference on Computer Communications.

[35] Ljung L. (1987). System Identification: Theory for the User.

[36] GLPK (GNU Linear Programming Kit). https://www.gnu.org/software/glpk/.

[37] Beloglazov A., Buyya R., Lee Y.C., Zomaya A. (2011). A taxonomy and survey of energy-efficient data centers and cloud computing systems. Advances in Computers.

[38] Falkner M., Leivadeas A., Lambadaris I., Kesidis G. Performance analysis of virtualized network functions on virtualized systems architectures. Proceedings of the IEEE CAMAD 2016-21st IEEE International Workshop on Computer Aided Modelling and Design of Communication Links and Networks.

[39] Bohnert F., Zukerman I. (2014). Personalised viewing-time prediction in museums. User Model.-User-Adapt. Interact..

[40] Dash S. (2005). Exponential lower bounds on the lengths of some classes of branch-and-cut proofs. Math. Oper. Res..

[41] Kindermann R., Snell J.L. (1980). Markov random fields and their applications. Am. Math. Soc..

[42] Geman S., Geman D. (1984). Stochastic relaxation, Gibbs distributions, and the Bayesian restoration of images. IEEE Trans. Pattern Anal. Mach. Intell..

[43] Silva Filho M.C., Oliveira R.L., Monteiro C.C., Inácio P.R., Freire M.M. CloudSim plus: A cloud computing simulation framework pursuing software engineering principles for improved modularity, extensibility and correctness. Proceedings of the IFIP/IEEE IM 2017—The 15th IFIP/IEEE International Symposium on Integrated Network Management.

[44] Cao J., Zhao Y., Lai X., Ong M.E.H., Yin C., Koh Z.X., Liu N. (2015). Landmark recognition with sparse representation classification and extreme learning machine. J. Frankl. Inst..

[45] Chen Z., Hu W., Wang J., Zhao S., Amos B., Wu G., Ha K., Elgazzar K., Pillai P., Klatzky R. An empirical study of latency in an emerging class of edge computing applications for wearable cognitive assistance. Proceedings of the SEC ’17—The Second ACM/IEEE Symposium on Edge Computing.

[46] Le Tan C.N., Klein C., Elmroth E. Location-aware load prediction in edge data centers. Proceedings of the IEEE FMEC 2017-The 2nd International Conference on Fog and Mobile Edge Computing.

[47] Jin C., Bai X., Yang C., Mao W., Xu X. (2020). A review of power consumption models of servers in data centers. Appl. Energy.

